# Synergistic cooperation between ABT-263 and MEK1/2 inhibitor: effect on apoptosis and proliferation of acute myeloid leukemia cells

**DOI:** 10.18632/oncotarget.6417

**Published:** 2015-11-27

**Authors:** Kelly Airiau, Valérie Prouzet-Mauléon, Benoit Rousseau, Arnaud Pigneux, Marie Jeanneteau, Manon Giraudon, Kaoutar Allou, Pierre Dubus, Francis Belloc, François-Xavier Mahon

**Affiliations:** ^1^ Laboratoire d'Hématopoïèse Leucémique et Cibles Thérapeutiques, INSERM 1035, Université Bordeaux, Bordeaux, France; ^2^ CHU Bordeaux, Hôpital Haut-Lévêque, Laboratoire d'Hématologie, Pessac, France; ^3^ Animalerie A2, Université Bordeaux Segalen, Bordeaux, France; ^4^ EA 2406 Histologie et Pathologie, Université Bordeaux, Bordeaux, France

**Keywords:** acute myeloid leukemia, MEK inhibitors, BH3 mimetic drugs

## Abstract

In spite of intensive research to improve treatment of acute myeloid leukemia (AML) more than half of all patients continue to develop a refractory disease. Therefore there is need to improve AML treatment. The overexpression of the BCL-2 family anti-apoptotic members, like BCL-2 or BCL-xL has been largely reported in lymphoid tumors but also in AML and other tumors. To counteract the anti-apoptotic effect of BCL-2, BH3 mimetics have been developed to target cancer cells. An increase in activity of ERK1/2 mitogen activated protein (MAP) kinase has also been reported in AML and might be targeted by MEK1/2 inhibitors. Hence, in the current work, we investigated whether the association of a BH3 mimetic such ABT-263 and the MEK1/2 inhibitor pimasertib (MEKI), was efficient to target AML cells. A synergistic increasing of apoptosis was observed in AML cell lines and in primary cells without affecting normal bone marrow cells. Such cooperation was confirmed on tumor growth in a mouse xenograft model of AML. In addition we demonstrated that MEKI sensitized the cells to apoptosis through its ability to promote a G1 cell cycle arrest. So, this combination of a MAP Kinase pathway inhibitor and a BH3 mimetic could be a promising strategy to improve the treatment of AML.

## INTRODUCTION

Acute myeloid leukemia (AML) is the commonest type of acute leukemia in adults, and is characterized by an abnormal proliferation, without differentiation, of myeloid precursors [1]. Currently, intensive chemotherapy is used as induction treatment allowing complete remission rates varying between 70 and 80% in patients younger than 60 years. However, most of these patients relapse and the 5-year survival ranges from only 30–40% [2]. Prognosis is worst for elderly patients, partly due to unfavorable cytogenetic abnormalities [3] and increasing frequency of resistance mechanisms [4]. Moreover, aggressive induction treatments have been associated with higher morbidity in these patients [5, 6]. Thus, there is a pressing need for new therapeutic strategies for AML to be developed.

Targeting anti-apoptotic proteins of the BCL-2 family is one such potential therapeutic possibility. These proteins play a key role in regulating apoptosis at the mitochondrial level. They contain at least one of the four BCL-2 homology domains (BH1, BH2, BH3 and BH4) and are divided into three groups: the anti-apoptotic proteins such as BCL-2, BCL-xL or MCL-1 interacting with and inhibiting the two sub-classes of pro-apoptotic proteins, the effectors BAX and BAK, and the BH3-only proteins like BIM or BAD [7]. BAX and BAK are activated when the pro-/anti-apoptotic balance is committed toward cell death, and are responsible for mitochondrial outer membrane permeabilization (MOMP) followed by caspase activation [8]. A high expression level of BCL-2, MCL-1 or BCL-xL has been reported in AML and associated with poor prognosis [9–11].

The BCL-2 family proteins are frequently associated with treatment resistance and are attractive targets for the development of anti-cancer agents [12, 13]. ABT-737 is a synthetic drug designed to mimic the BH3 domain of BAD and is consequently an effective inhibitor of BCL-2 and BCL-xL. We have previously demonstrated that ABT-737 cooperates with tyrosine kinase inhibitors (TKI) towards BIM stabilization to increase apoptosis in chronic myeloid leukemia (CML) cells [14, 15].

BIM expression is highly regulated at the post-translational level. Extracellular signal-regulated kinases 1/2 (ERK1/2) mediate BIM phosphorylation on serine 69, promoting its degradation via the proteasome pathway [16]. ERK1/2 kinases belong to the mitogen activated protein kinase (MAPK) pathway and are downstream effectors of MEK1/2. An activation of the RAS/RAF/MEK/ERK pathway has been observed in many tumors, including AML, leading to the development of specific inhibitors of this pathway [17–19]. Several studies have reported that MEK inhibitors (MEKI) can induce apoptosis through BIM accumulation [20–22].

Orally bioavailable MEKI and BH3 mimetics are currently tested in clinical trials as single treatment and we aimed to investigate the effectiveness of two of these molecules used in combination, i. e. AS703026 (pimasertib) and ABT-263. We observed a synergistic cooperation between drugs in AML cell lines *in vitro* and in a xenograft model. The blockage in G1 phase induced by MEKI, seems to be mandatory to improve apoptotic response to ABT-263. The beneficial effect of MEKI/ABT-263 association was confirmed in leukemic progenitors cells from AML patients.

## RESULTS

### ABT-263 and MEKI are synergistic in leukemic cells lines

**Figure 1:**
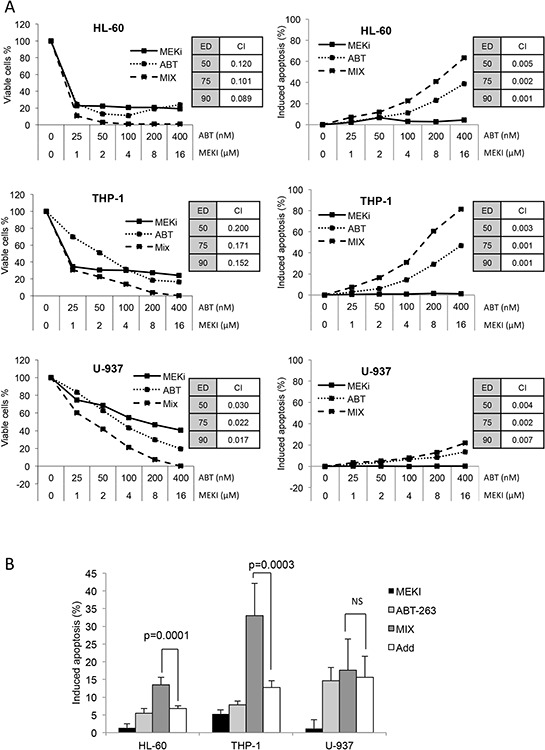

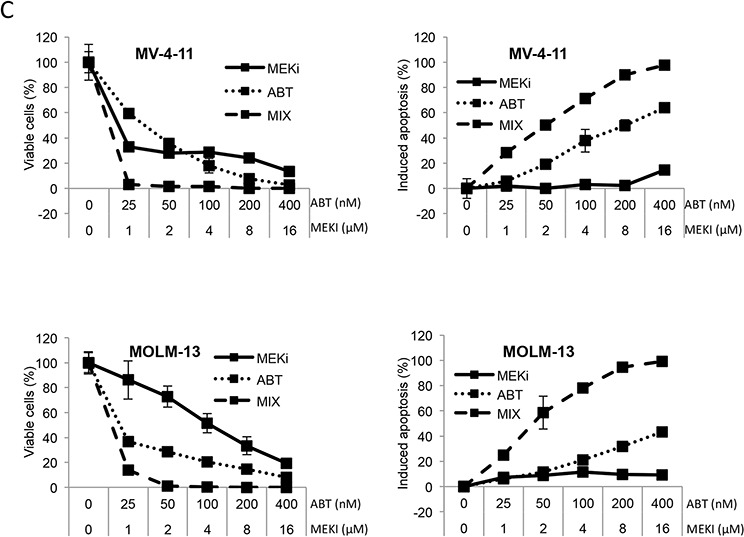
MEKI and ABT-263 synergize to inhibit cell proliferation and induce apoptosis in AML cell lines **A.** and **C.** HL-60, THP-1 or U-937 (A) and MV-4–11 or MOLM-13 (C) cells were incubated with increasing concentrations of ABT-263, MEKI or both at a constant ratio (Mix). The amount of viable cells was measured through their ATP content by chemoluminescence after a 72-h culture (left column). The percentage of apoptotic cells was determined by flow cytometry with a MMP probe after a 24-h incubation (right column). Combination indexes were calculated at ED 50, 75 and 90 using the Calcusyn software. **B.** HL-60, THP-1 or U-937 cells were treated for 24 h with 1 μM MEKI (black bars), 200 nM ABT-263 (light grey bars) or both (MIX, grey bars). Apoptosis was then measured by flow cytometry as described in Figure 1. Mean +/− SD of seven experiments is shown. The calculated additive effect of both drugs is also shown (white bars) and compared to the measured MIX effect using the Student paired *t* test. **C.** MV-4–11 or MOLM-13 (C) cells were incubated with increasing concentrations of ABT-263, MEKI or both at a constant ratio (Mix). The amount of viable cells was measured through their ATP content by chemoluminescence after a 72-h culture (left column). The percentage of apoptotic cells was determined by flow cytometry with a MMP probe after a 24-h incubation (right column).

Three cell lines (HL-60, THP-1 and U-937 cells) were used in this study as *in vitro* AML cell models, to investigate the effect of ABT-263 and MEKI used in combination. Both ABT-263 and MEKI produced a dose-dependent inhibition of cell proliferation (Figure 1-A, left column). However, unlike ABT-263, MEKI alone did not induce apoptosis (Figure 1-A, right column). In combination, we observed a synergistic effect to inhibit cell proliferation in the 3 AML cell lines. Considering induced-apoptosis, the synergistic effect was confirmed in HL-60 (*p* = 0.0001) and THP-1 (*p* = 0.0003) and moderate in U-937 cells (Figure 1B).

FLT3-ITD mutation is the best-known molecular abnormality of AML, so we used two FLT-3-ITD positive cell lines: MV-4–11 and MOLM-13. A synergistic effect between MEKI and ABT-263 was also obtained on these two specific cell liens (Figure 1C). Considering these results, we choose to follow our investigations on HL-60, THP-1 and U-937 cells

### Expression of BCL2 family protein partially depends on ERK1/2 phosphorylation

**Figure 2:**
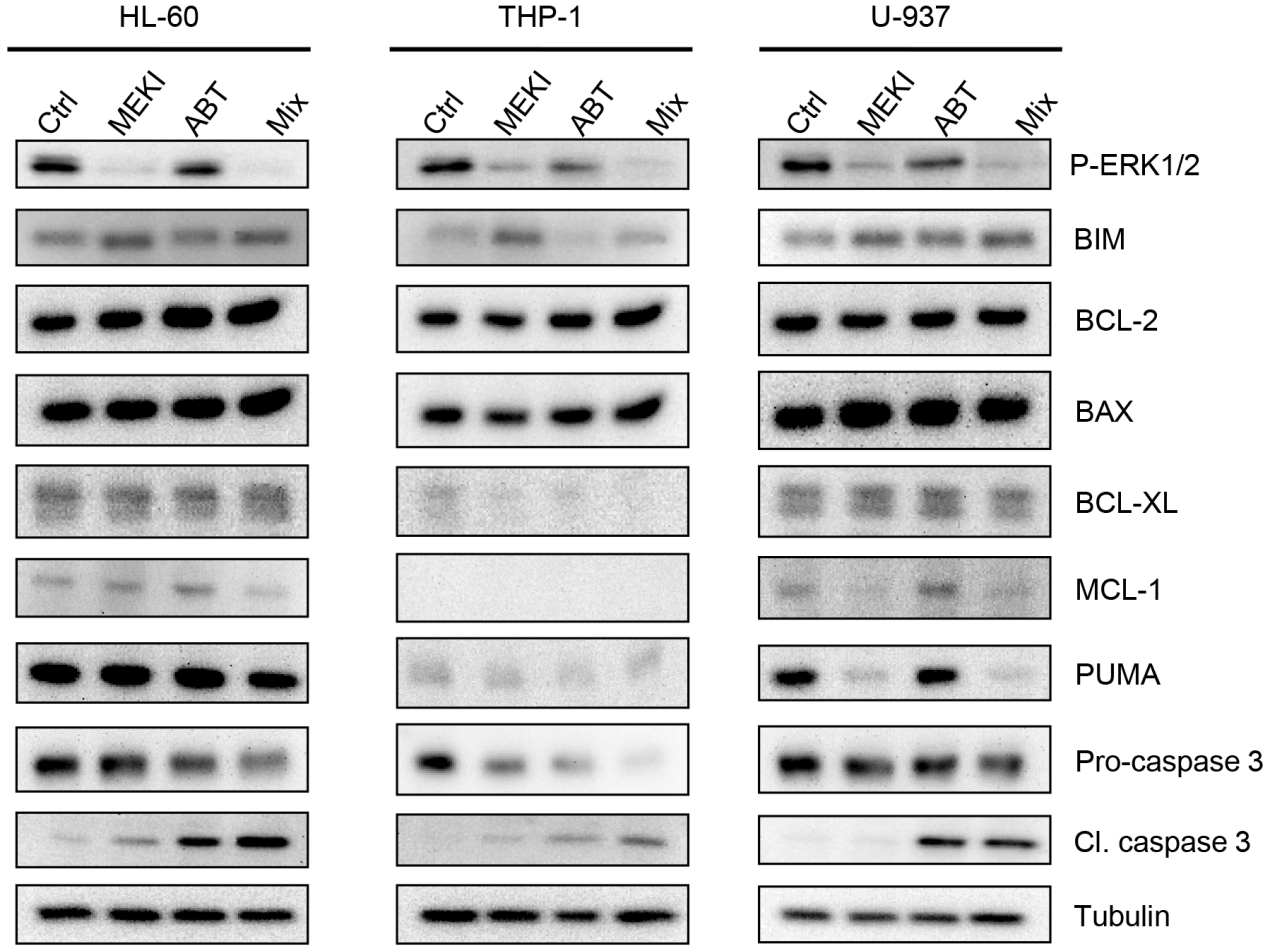
Protein expression of the Bcl-2 family members following ABT-263 and MEKI treatment HL-60, THP-1 or U-937 cells were analyzed by western blot for expression of the Bcl-2 family proteins. The three cell lines were treated for 24 h with 1 μM MEKI, 200 nM ABT-263 or both (Mix) and analyzed for Erk1/2 phosphorylation, BIM, Bcl-2, BAX, Bcl-XL, Mcl-1 and PUMA expression and Caspase 3 cleavage.

To investigate the heterogeneity in drug combination responses we analyzed the expression of BCL-2 family proteins in the 3 different cell lines. Indeed, the constitutive pro-/anti-apoptotic balance between the BCL-2 family proteins differed between the 3 cell lines, with high BCL-xL and PUMA expression in HL-60 and U-937 (Figure 2), but similar BCL-2 and BAX levels in the 3 cell lines. Furthermore, MCL-1, known as a resistance factor to ABT-263, was highly expressed in U-937 but also in the responsive HL-60 cells (Figure 2). MEKI addition resulted in a dephosphorylation of ERK1/2 and was accompanied by an increase in BIM protein content. This BIM accumulation was not sufficient to induce the cleavage/activation of caspase 3 by itself (Figure 2). However, it increased the rate of caspase 3 cleavage when the cells were treated with ABT-263 in HL-60 and THP-1 but not in U-937 cells. MEKI also induced a decrease in PUMA level in U-937 but not in HL-60 while THP-1 did not expressed PUMA, MCL-1 nor BCL-xL.

### The synergistic cooperation between ABT-263 and MEKI is partially linked to BIM accumulation

**Figure 3:**
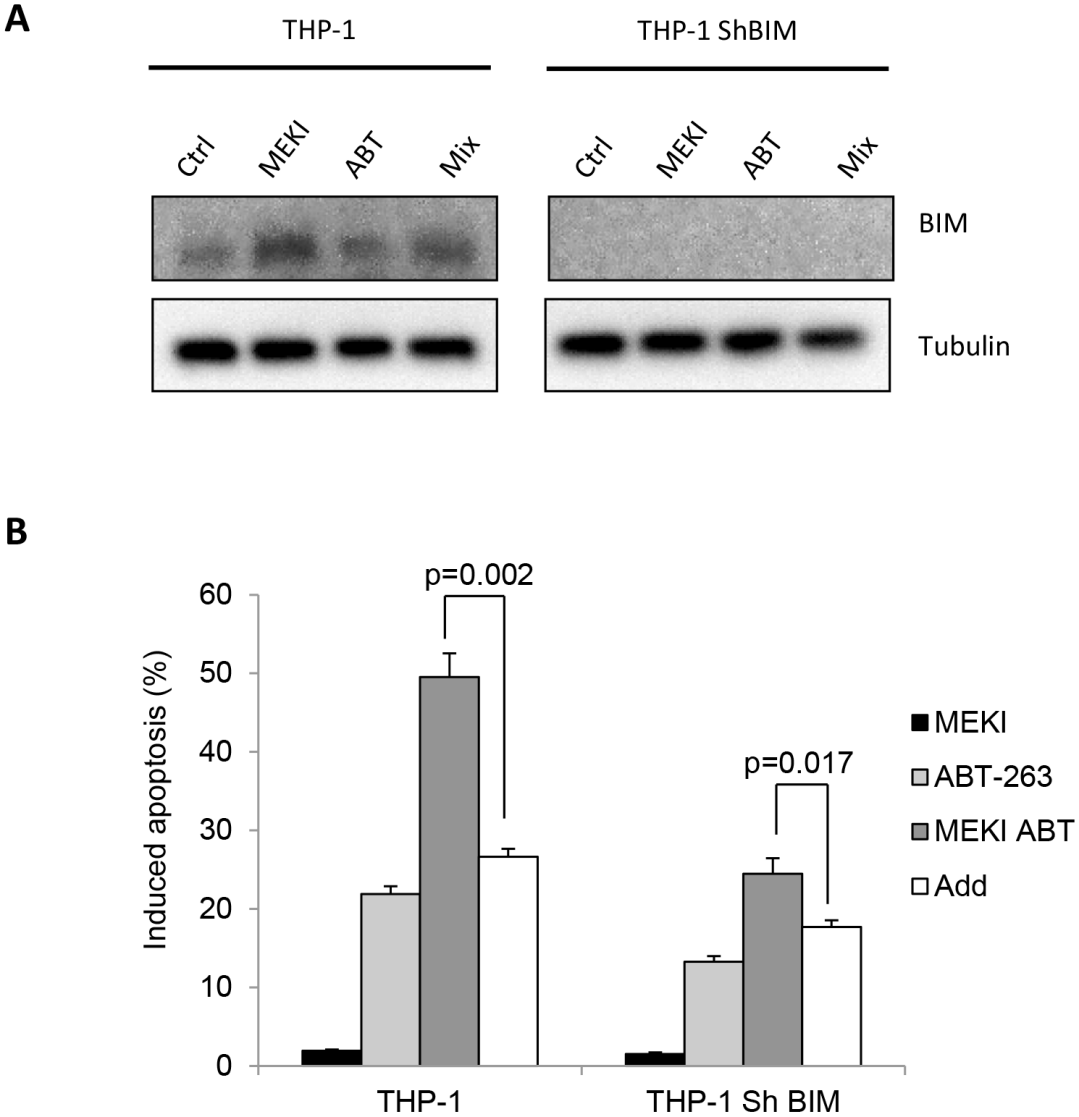
BIM accumulation does not participate in the cooperation between ABT-263 and MEKI to induce apoptosis in THP-1 THP-1 cells or THP-1 cells depleted for BIM by RNA interference were treated with 1 μM MEKI, 200 nM ABT-263 or both in combination (Mix) for 24 h and analyzed for BIM expression **A.** and apoptosis induction **B.**

To investigate the role of BIM accumulation in the synergistic effect of the two drugs in THP-1 cells, we developed a BIM-deficient THP-1 cell line by using RNA interference (Figure 3A). Although responding to ABT-263 to a lesser extent (about 50% less) than wild type THP-1, BIM-deficient cells synergistically responded to ABT-263 and MEKI co-treatment (Figure 3B). (*p* = 0.001). Thus, if BIM accumulation contributed to the cumulative drug-induced apoptosis in THP-1 cells, it was not sufficient to explain the synergistic cooperation between ABT-263 and MEKI.

### Apoptotic response to ABT-263 depends on the blockage in G1 phase induced by MEKI

**Figure 4:**
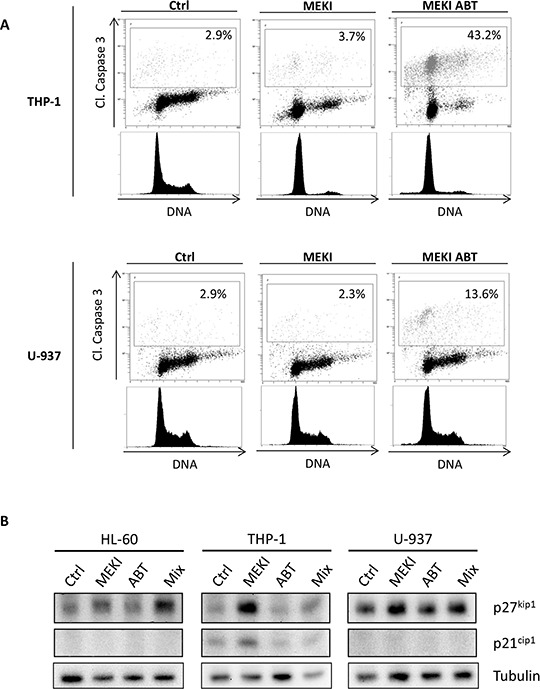
ABT-263-induced apoptosis depends on the cell cycle block in G1 phase **A.** THP-1 and U-937 cells were untreated or treated with 1 μM MEKI or a combination of 1 μM MEKI and 200 nM ABT-263 for 24 h. Cells were then fixed, permeabilized and stained with anti-cleaved caspase 3 antibody (Y axis) and Vibrant Violet Cycle probe (X axis). Cells were then analyzed by flow cytometry. **B.** HL-60, THP-1 or U-937 cells were treated for 24 h with 1 μM MEKI, 200 nM ABT-263 or both (Mix) and analyzed by western blot for p27kip1, p21cip1 and tubulin expression.

**Figure 5:**
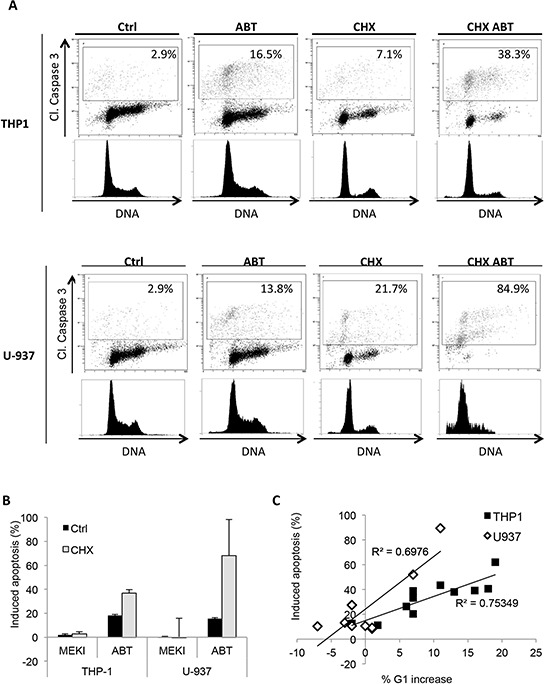
CHX induces a cell cycle block in G1 phase and synergizes with ABT-263 to increase apoptosis **A.** THP-1 and U-937 cells were pre-treated or not with 3 μM CHX for 24 h and treated or not with 200 nM ABT-263 for 24 h. Cells were then fixed, permeabilized and stained with anti-cleaved caspase 3 antibody (Y axis) and Vibrant Violet Cycle probe (X axis) and analyzed by flow cytometry. **B.** THP-1 and U-937 cells were pre-treated or not with 3 μM CHX for 24 h and treated or not with 1 μM MEKI, 200 nM ABT-263 or both (Mix) for 24 h. Cells were then fixed, permeabilized and stained with anti-cleaved caspase 3 antibody and analysed by flow cytometry. Mean +/− SD of three experiments is shown. **C.** THP-1 (black squares) and U-937 (white diamonds) cells were pre-treated with MEKI (200 nM) or CHX (3 μM) for 24 h. Samples were then treated or not with 200 nM ABT-263 for 24 h and analyzed by flow cytometry for cell cycle and caspase 3 activation. The graph represents the correlation between the percentage of ABT-263-induced apoptosis and the increased amount of cells in G1 phase.

The RAS/RAF/MEK/ERK signaling cascade plays a central role in regulating cell proliferation, differentiation or death. ERK1/2 involvement in the regulation of G1 to S phase transition has already been demonstrated [27, 28]. We observed that MEKI induced a G1 cycle block in the THP-1 cell line (Figure 4A), whereas ABT-263 induced apoptosis through caspase 3 cleavage in G1 phase (Supplementary Figure S1). When both molecules were used in combination, the ABT-263-induced increase in apoptosis was dependent on the block in G1 phase (Figure 4A). Such a MEKI-induced G1 block was not observed in U-937 cells (Figure 4A). To support our results, we investigated the effect of MEK inhibition on the expression of two proteins involved in G1/S phase transition: p27kip1 and p21cip1. By western blot analysis, we observed a p27kip1 accumulation in HL-60 and THP-1 but not in U-937 cells and an increase in p21cip1, only expressed by THP-1, after MEK inhibition. These results suggested that the synergistic cooperation between ABT-263 and MEKI relied on a MEKI ability to block cells in G1 phase. Thus, we tested the ability of cycloheximide (CHX), a known G1 blocker [29], to cooperate with ABT-263. We confirmed that CHX treatment induced a block in G1 phase in both THP-1 and U-937 cells (Figure 5A). This was associated with a dramatic increase in ABT-263-induced apoptosis in both cell lines (Figure 5B). We also observed a positive correlation between MEKI- or CHX-induced increase in G1 arrest and ABT-263-induced apoptosis in THP-1 cells (Figure 5C, black squares). Such a correlation was verified in U-937 cells treated by CHX while MEKI was not able to increase the ABT-263 effect due to the absence of block in G1 (Figure 5C, white diamonds). These results confirmed the importance of a G1 cell cycle arrest to promote ABT-263-induced apoptosis.

### The synergistic inhibition effect of ABT-263 and MEKI on primary leukemic cells

**Figure 6:**
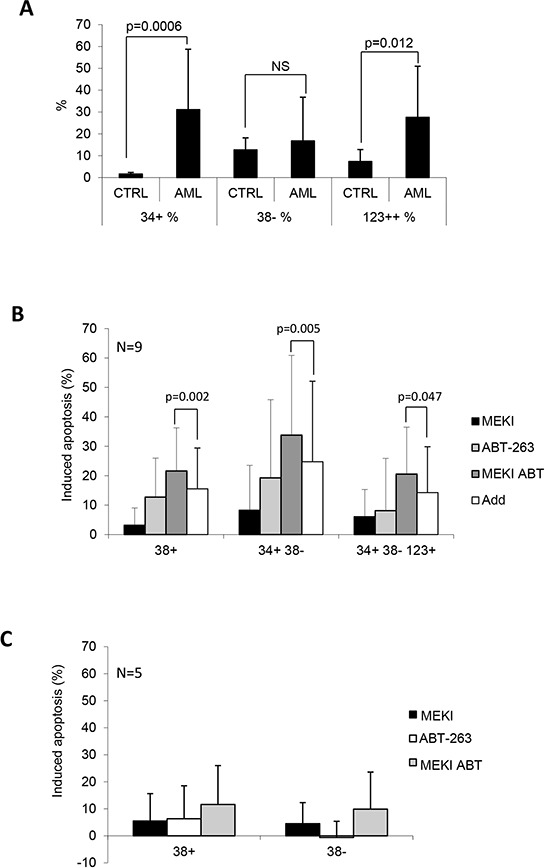
MEKI and ABT-263 synergize to induce apoptosis in leukemic stem cells Mononuclear cells from AML or normal control bone marrows were labelled with APC-CD34, PC5-CD38, PE-CD123 and FITC-annexin V. **A.** The percentage of CD34+ cells, CD38- cells in the CD34+ population and CD123+ cells in the CD34+/CD38- population was plotted for normal control and AML samples. Results are expressed as mean +/–SD and are representative of 10 and 17 experiments for respectively control and AML bone marrows. P indicates the significance using the Mann-Whitney test. Annexin-V+ cells were considered as apoptotic and their percentage was evaluated in the CD34+/CD38+, CD34+/CD38-, and CD34+/CD38-/CD123+ populations. **B.** Apoptosis induction was evaluated in 9 AML samples as indicated in each of the three populations after treatment with MEKI (black bars), ABT-263 (white bars) or both (grey bars). C: Apoptosis induction was evaluated in 6 normal control samples in the CD34+/CD38+ and CD34+/CD38- populations.

We verified the effect of these drugs on primary normal (*n* = 6) and leukemic (*n* = 9) bone marrow MNC. These were labelled with anti-CD34, anti-CD38 and anti-CD123 (Supplementary Figure S2) and analyzed by flow cytometry for their ability to bind annexin V after treatment with either 20 nM ABT-263, 1 μM MEKI or both in combination (Figure 6). As expected, AML samples were enriched in CD34 expressing cells (leukemic blast cells) as compared to normal bone marrow MNC (Figure 6A). Among the CD34+ cells, the rate of CD38- cells did not differ between normal and leukemic samples, but leukemic CD34+/38− cells were enriched for CD123+ cells (Figure 6A, *p* = 0.012), as previously described for the so-called leukemic stem cells [30]. MEKI alone was poorly pro-apoptotic in the populations analyzed (Figure 6B). At 20 nM ABT-263 induced a weak apoptosis rate in CD34+ cells but not in the CD34+/CD38-/CD123+ population. In comparison to the untreated condition, the combination of MEKI and ABT-263 induced a significant apoptosis in both CD34+ (*p* = 0.001) and CD34+/CD38-/CD123+ leukemic stem cells (*p* = 0.045) (Figure 6B). Moreover, the apoptosis rate induced by the drug combination was greater than the addition of the separated effects of each drug meaning a synergistic effect (*p* = 0.002 for CD38+, *p* = 0.005 for CD38- and *p* = 0.047 for CD38-/CD123+). Under the same conditions, no significant apoptosis was induced in CD34+ normal bone marrow cells (Figure 6C).

### MEKI enhances the therapeutic efficacy of ABT-263 in a murine model of human AML

**Figure 7:**
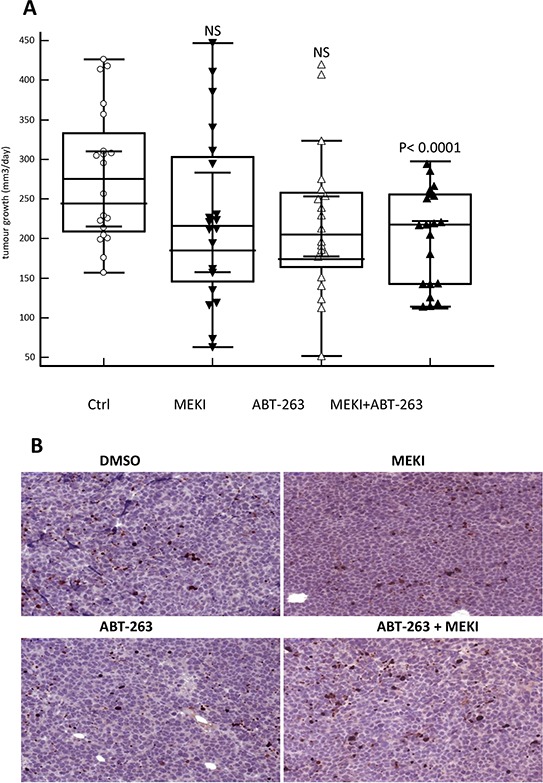
MEKI and ABT-263 synergize to reduce tumor growth *in vivo* **A.** HL60 cells were subcutaneously injected in NSG mice and mice were treated five days a week with vehicle control, MEKI (0.2 mg/kg), ABT-263 (1 mg/kg) or both. The growth rate was calculated from the measurement of the tumour volume twice a week and defined as the increase in mm3/day in each mouse. The graph represents the values of two independent experiments performed on 10 mice/group for each experiment. **B.** Histological sections of tumours stained with anti-activated caspase 3 antibody. A significant field of slides in tumours from either vehicle-, MEKI-, ABT-263- or MEKI + ABT-263-fed mice is shown as indicated.

To further validate the beneficial association between MEKI and ABT-263, *in vivo* experiments were performed. Drug effect was investigated on the growth of tumour issued from HL-60 cell line subcutaneously implanted in NSG mice. Mice were orally treated with low doses of MEKI, ABT-263 or both in combination. All mice were alive and in good fitness at the end of the experiments after three weeks treatment. While each inhibitor separately was unable to significantly decrease the rate of tumour growth, their combination induced a highly significant 27% decrease in growth rate (*p* < 0.0001, Mann-Whitney test) (Figure 7A). At the end of the experiments, the weight of the tumours was decreased by 21% in the combination treatment group (2.05+/–0.70g versus 2.60+/–0.97g, *p* = 0.047, Student *t* test) as compared to the vehicle group while no significant decrease was observed in the single drug groups. Moreover, the drug combination induced an increase in the frequency of apoptotic cells inside the tumour as compared to vehicle or individual drug treatment (Figure 7B). In parallel, we observed that MEKI treatment alone or associated with ABT-263 induced a decrease in the phosphor-ERK1/2 staining *in vivo* (Supplementary Figure S3).

## DISCUSSION

We investigated the effect of the BH3 mimetic ABT-263 used in combination with the MEK1/2 inhibitor AS703026, also known as pimasertib, on AML cells. Both molecules are currently being tested in clinical trials as single drug, in phase II for ABT-263 (NCT00445198) [31] and in phase II for AS703026 (Study NCT00957580). *In vitro*, we observed in combination a synergistic effect in apoptosis induction in HL-60 and THP-1 cell lines but not in U-937 cells. This heterogeneity in response rates could be partly explained by the differential expression of the BCL-2 family members or by the inability of the MEKI to block the cell cycle in G1 phase in U-937 cells. We need to take into account that AML is a heterogeneous disease depending on many factors such as blast cells number, lineage, genetic events and stromal environment. The mutational status could also be take into account. FLT3-ITD mutation is observed in 25% of AML. Here, we showed that FLT-ITD mutation doesn't modify MEKI or ABT-263 responses. Despite this heterogeneity, we confirmed the results on primary cells from AML patients and observed a significant increase in apoptosis of leukemic progenitors and stem cells while there was no effect on normal cells.

We previously reported a synergistic cooperation between a BH3 mimetic and BIM stabilization by TKI in CML cells [14]. As compared to CML, in AML cells BIM expression is regulated by post-translational modifications, including its phosphorylation by ERK1/2, resulting in its degradation by the proteasome [16]. We thus wondered if ERK1/2 inhibition could promote the ABT-263-induced apoptosis in AML cells. As expected, MEK1/2 inhibition resulted in a significant decrease in ERK1/2 phosphorylation that was associated with BIM accumulation. However, this BIM accumulation did not result in caspase 3 cleavage by itself. When MEKI was associated with the BH3 mimetic, the BIM accumulation was linked to an increased apoptosis in only two of the three cell lines tested. Moreover, RNA interference experiments showed that the loss of BIM accumulation reduced apoptotic rated but didn't impair MEKI/ABT-263 cooperation. These results showed that while BIM accumulation drive ABT-263-induced apoptosis, it is not necessary for a synergistic cooperation between ABT-263 and MEKI, suggesting that other mechanisms could be involved. Konopleva et al. [22] have reported that MEKI cooperates with ABT-737 to increase apoptosis in the OCI-AML3 cell line, through MCL-1 down-regulation and enhanced BIM binding to MCL-1. In our work, we also observed a MEKI-induced MCL-1 down regulation in U-937 but this was not associated with a synergistic response to the combination treatment. In parallel, no MCL-1 decrease was evidenced on HL-60 and THP-1 cells which were deficient for MCL-1 (see Figure 2). This deficiency could explain the very strong synergism observed in THP-1, however, those cells were not more sensitive to ABT-263 alone than the two others. And, in spite of a high MCL-1expression in U-937, ABT-263-induced apoptosis was not weaker than in HL-60 or THP-1 (Figure 2A). It is possible that apoptosis mechanism was independent of BCL2 in U-937 as recently reported [32]. Besides, MCL-1 was also strongly expressed in HL-60 showing that it did not interfere with the synergy. The discrepancy between our work and the previous study [22] can be explained by the different MEKI or types of cell lines used, and suggests that mechanisms other than the pro-/anti-apoptotic balance between the BCL-2 family members are implicated in this synergy.

ERK1/2 involvement in G1/S transition has been previously demonstrated (2728). In this study, we confirmed that MEK1/2 inhibition promoted a G1 cell cycle arrest in THP-1 cells that was associated with the accumulation of two cell cycle inhibitors: p27kip1 and p21cip1. In parallel, we showed that ABT-263 induced apoptosis in G1 phase without affecting the cell cycle distribution. Thus, by inducing a G1 block, MEKI could facilitate the apoptotic response to ABT-263 treatment, resulting in a synergistic cooperation. In U-937 cells that were resistant to MEKI-inhibited cell cycle progression. The cause of the MEKI inefficiency to block U-937 cells in G1 phase is not known. When treating U-937 and THP-1 cells with CHX, a protein synthesis inhibitor known to be able to arrest the cell cycle at the transition point in G1 phase, we observed effective G1 arrest in both cell lines, with a consequent synergistically cooperation with ABT-263 to increase apoptosis. The CHX-induced G1 block is higher in U-937 than in THP-1 and this correlates with apoptotic response obtained after CHX/ABT-263 treatment. This G1-dependant apoptosis does not depend on p53 as the three cell lines we used in this work were deficient for p53. These results highlight the importance of the cell cycle phase besides the BCL-2 family member status for ABT-263 efficiency in inducing apoptosis. The levels of expression or phosphorylation of the BCL-2 family proteins could make them in a less stable equilibrium during the G1 phase facilitating the pro-apoptotic effect of ABT-263. Alternatively, the levels of ROS in G1 cells as compared to S/G2M cells could sensitize the cells to BCL-2 inhibition as described for leukemic stem cells [33].

Interestingly, the combination of MEKI and very low ABT-263 concentrations (20 nM) was effective in inducing apoptosis in both leukemic blast (CD34+/CD38+) and stem (CD34+/CD38–/CD123+) cells. These results confirmed those previously described for ABT-737 and PD0325901 or CI1040 [22]. Importantly, the combination of MEKI and ABT-263, at the weak concentrations we used here, was ineffective on normal bone marrow CD34+ cells and could thus be a promising therapeutic approach to target leukemic cells without affecting normal bone marrow progenitor cells.

Consequently, *in vivo* experiments were performed on a xenograft mouse model to test the cooperation between both inhibitors. The drugs dosing with ABT-263 and MEKI was lower than in previous reports to avoid all side effects and maintaining drugs concentration in the range of specific inhibition of their targets [34–37]. Even in these restricted conditions, the beneficial of the MEKI/ABT-263 combination was evidenced on the growth of a particularly study tumour model i.e. subcutaneous HL-60 implant. This confirms that combining the specific effects of each drugs can be envisaged *in vivo* at a dosing lower than the efficient dose of each drug thus avoiding the adverse effects observed when each were separately used.

Despite the recent convincing demonstration of the efficiency of a selective BCL2 inhibition by ABT-199 in AML cells [38], a strategy using combining therapy with lower doses is probably the best way to overcome resistance and avoid side effects

Our overall results show that ABT-263 is more effective when used in combination therapies. A better understanding of the mechanisms responsible for the resistance of the U-937 cell line could allow determining which patients could benefit from such a therapeutic strategy. However, among the primary AML cells analyzed in this study, no sample was resistant *in vitro* to the combination therapy. Considering these results, ABT-263 and MEKI combination seems to be a very promising strategy and should be proposed to treat AML patients in a phase 2 study.

## MATERIAL AND METHODS

### Cell lines

HL-60, THP-1, U-937, MV-4–11 and MOLM-13 cell lines were cultured in RPMI 1640 (Gibco, Saint-Aubain, France) supplemented with 10% v/v fetal calf serum (FCS), 1 mM glutamine, 25 mM Hepes, 100 units/ml penicillin, 50 μg/ml streptomycin in a humidified atmosphere containing 5% v/v CO2 at 37°C. Exponentially growing cells were used in all experiments.

Down-regulation of BIM expression was achieved through lentiviral expression of siRNAs. THP-1 cells were transduced with lentiviral vectors (C-shRNA and BIM-shRNA) at a multiplicity of infection of 2 in RPMI supplemented with 10% FCS and 8 μg/mL protamine sulfate (Sigma, St Louis, MI). Transduction efficiency was verified by testing the EGFP expression by flow cytometry. After one week of culture, GFP-expressing cells were sorted by flow cytometry using an Elite-EPICS cell sorter (Beckman-Coulter, Villepinte, France). At the time of the experiments, more than 95% of cells were fluorescent [23].

### Bone marrow samples

Bone marrow samples were obtained from patients with AML and from non-hematological patients (‘normal’) under informed consent obtained in accordance with the Declaration of Helsinki from all participants and data were analyzed anonymously. The study was approved by the local Ethics at the University of Bordeaux. Mononuclear cells (MNC) were separated by Ficoll sedimentation (PAN Biotech GmbH, Germany), washed and suspended in culture medium in the presence of the different drugs.

### Reagents

ABT-263 (Navitoclax^®^, Abbott) and AS703026 (Merck Serono) were purchased from Selleck Chemicals LLC (Houston, TX, USA). ABT-263 was used at 200 nM on cell lines and 20 nM on MNC. The AS703026 notated MEKI on the text, was used at 1 μM. Stem α4B (Stem Cell Technology, Grenoble, France), a cytokine cocktail containing IL-3, IL-6, GM-CSF, EPO, TPO and SCF, was used at 100 μL/mL. Cycloheximide (CHX) (Calbiochem, Fontenay-sous-bois, France) was used at 3 μM.

### Apoptosis in cell lines

Apoptosis was detected by using DiOC6(3) (100 ng/mL) as a probe for mitochondrial membrane potential (MMP), as previously described [24]. For cell cycle analysis, cells were fixed with 4% formaldehyde for 10 min at room temperature, permeabilized with 0.1% Triton X100 for 10 min at 37°C, and post-fixed in 50% methanol at 4°C for 10 min. They were then washed and rehydrated with 3% bovine serum albumin in PBS, labeled with a primary rabbit anti-cleaved caspase 3 monoclonal antibody (Cell Signalling, Danvers, United States) and a secondary anti-rabbit IgG antibody coupled with Alexa Fluor 647 (Molecular Probes, Invitrogen, Saint-Aubin, France) and stained for DNA content using Violet Vibrant DyeCycle (Molecular Probes, Invitrogen). Analysis was performed on a Navios cytometer (Beckman Coulter) with red and violet excitations. Cleaved caspase 3 was measured at 660 nm and DNA content at 450 nm.

### Apoptosis in primary bone marrow cells

After a 24-h incubation with the indicated drugs, MNC from AML patient or normal bone marrows were labeled with anti-CD34-APC, anti-CD38-PC5, anti-CD123-PE and Annexin V-FITC. Samples were analyzed by multivariate flow cytometry (Navios, Beckman Coulter). Percentages of annexin V-positive cells were quantified on the gated CD34+/CD38+ or CD34+/CD38- or CD34+/CD38-/CD123+ cells, and considered as apoptotic.

### Cell proliferation

Cells were seeded at 25,000 per well in a 96-well plate and cultured for 3 days with increasing concentrations of MEKI, ABT-263 or both, in combination. Viable cells were evaluated on the basis of their ATP content. The CellTiter-Glo^®^ luminescent viability assay (Promega, Madison, United-States) was used according to the supplier's instructions. The bioluminescence was read using a Wallac 1420 Victor luminometer.

### Western Blot

After SDS-PAGE electrophoresis, proteins were transferred onto a PVDF membrane (Biorad, Marnes-la-Coquette, France). Membranes were saturated with 5% (w/v) fat-free dry milk or 5% (w/v) bovine albumin in Tris-buffered saline containing 0.1% (v/v) Tween 20 (Sigma). Membranes were then probed with primary antibodies: mouse monoclonal for BCL-2 (Sigma), BCL-xL (Santa Cruz Biotech.), and MCL-1 (BioLegend), rabbit monoclonal for caspase 3, cleaved caspase 3, phospho-p44/42 MAPK (Erk1/2) (Thr202/204), PUMA, p27kip1 and p21cip1 (Cell Signaling Technology, Inc, Danvers), and rabbit polyclonal for BAX (Santa Cruz), BIM and Tubulin (Sigma). All antibodies were used at a 1/1000 dilution. After secondary antibody labeling, peroxidase activity was revealed using the Western Lightning Plus-ECL kit (Perkin Elmer, Courtaboeuf, France) and band intensity was quantified using a Kodak Imager.

### Mouse xenograft models

Two sets of experiments were conducted, each with 40 NSG (NOD.Cg-Prkdcscid Il2rgtm1Wjl/SzJ) mice [25]. Mice were bred under specific pathogen-free conditions and experiments were performed in the “Service des animaleries” of the University of Bordeaux in conformity with the rules of the Institutional Animal Care and Use committee (approval number 03455.01). In the first series of experiment, 106 HL-60 cells were inoculated subcutaneously. In the second one, 2×105 cells only were injected. Tumour volume was measured and calculated with the formula length × width2/2. When average tumour size approximately reached 450 mm3 in the first set and 150 mm3 in the second one, the mice were randomized into control and three treated groups, of ten mice each. Mice were administered by oral gavages using a stomach tube with vehicle control (DMSO), ABT-263 (1 mg/kg), MEKI (0,2 mg/kg) or both. Mice were treated five days a week during three weeks. Tumour size was measured twice a week. At the end of the experiments, mice were sacrified by cervical dislocation and tumour was excised and weighted. The tumour growth was calculated in mm3/days as the slope of the regression line of the size of the tumour as a function of the duration of treatment for each mice. Both series of experiments were pooled in the statistical analysis and the significance between the treated and control groups were analyzed using the Mann-Whitney test using the Medcalc software.

### Apotosis and ERK1/2 activity in Xenograft models

Pieces of the tumours were fixed for 12 h in 3.7% formaldehyde in PBS, followed by 48 h in 70% Ethanol. Standard histological processing and paraffin embedding were done. Sections of 3 μm thickness from paraffin-embedded tissues (PET) were heated to 56°C, rehydrated, washed and treated with TE (Tris 10 mM, EDTA 1 mM pH9) 30 min at 98°C for antigen retrieval. Endogenous peroxidases were inhibited with 3% hydrogen peroxide (Sigma) in H2O for 5 min. Non specific sites and avidin were blocked with buffers from kit PK-7200 and SP-2001 from Vector laboratories (CA, USA). Slides were incubated with primary anti-Active caspase3 1:300 (AF835 R&D Systems Minneapolis, MN, USA) or anti-phospho-ERK1/2 (Cell Signaling) for 1 hour followed by 30 min incubation at room temperature with biotinylated secondary antibodies (SP-2001 Vector Laboratories) and revealed by an additional 10 min incubation with Novared (SK-4800 Vector Laboratories). Slides were counterstained with hematoxylin, dehydrated and mounted with Eukitt-mounting medium (Labonord).

### Statistical analysis

All experiments on cell lines were performed in triplicate and results expressed as the mean ± standard deviation (SD). The paired Student's *t*-test was used to analyze the data. Considering the elevated spontaneous apoptosis occurring during culture of patients' cells, drug-induced apoptosis was calculated as the percentage of drug specific apoptosis: (apoptosis in treated sample - apoptosis in control) × 100 divided by (100 - apoptosis in control). Experiments performed on patients' cells were analyzed for significance using either the Mann-Whitney or Wilcoxon test for paired samples.

The combination index (CI) was determined by the method of Chou-Talalay [26] using the Calcusyn software (Biosoft, Cambridge, UK). Results were expressed as the CI value at the effective dose inducing 50, 75 or 90% apoptosis (ED50, ED75 and ED90) for AML cell lines. A CI less than 1 is considered as synergistic effect, a CI equal to 1 is additive and a CI greater than 1 is antagonist.

## SUPPLEMENTARY FIGURES LEGENDS


