# Supplementary material for: Synergistic cooperation between ABT-263 and MEK1/2 inhibitor: effect on apoptosis and proliferation of acute myeloid leukemia cells
Source: Oncotarget. 2015 Nov 27;7(1):845–59. doi: 10.18632/oncotarget.6417 (PMC4808037; doi:10.18632/oncotarget.6417)
Supplement: Supplementary file 1 [file oncotarget-07-0845-s001.pdf]

SUPPLEMENTARY FIGURES LEGENDS

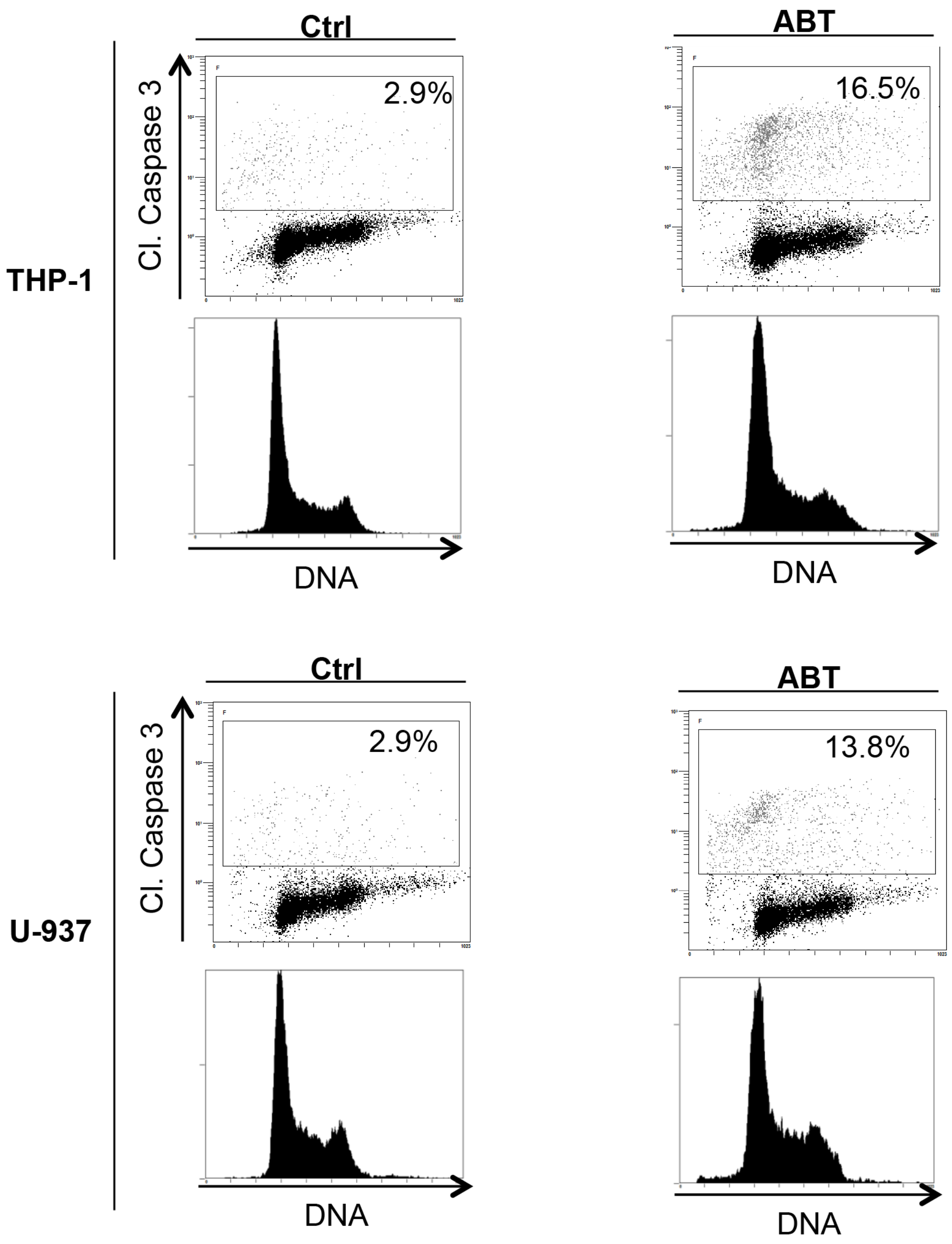

Supplementary Figure S1: ABT-263 induces apoptosis in G1 phase. THP-1 or U-937 cells were untreated or treated with 200 nM ABT-263 . They were then fixed, permeabilized and stained with anti-cleaved caspase 3 antibody (Y axis) and Vibrant Violet Cycle probe (X axis). Cells were then analyzed by flow cytometry.

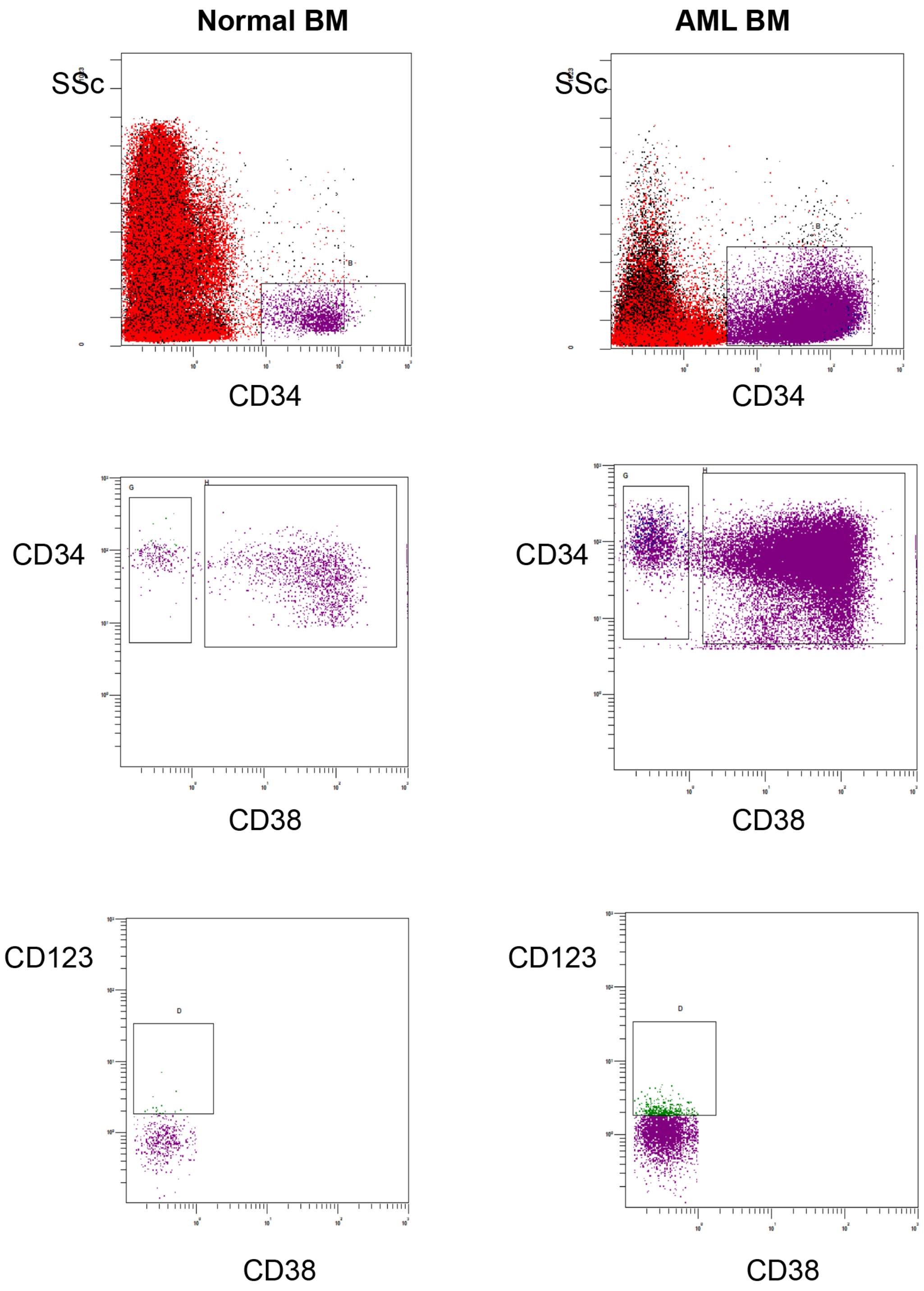

Supplementary Figure S2: Flow cytometry gating for CD34+ cell analysis in normal (left) and AML (right) bone marrow mononuclear cells.

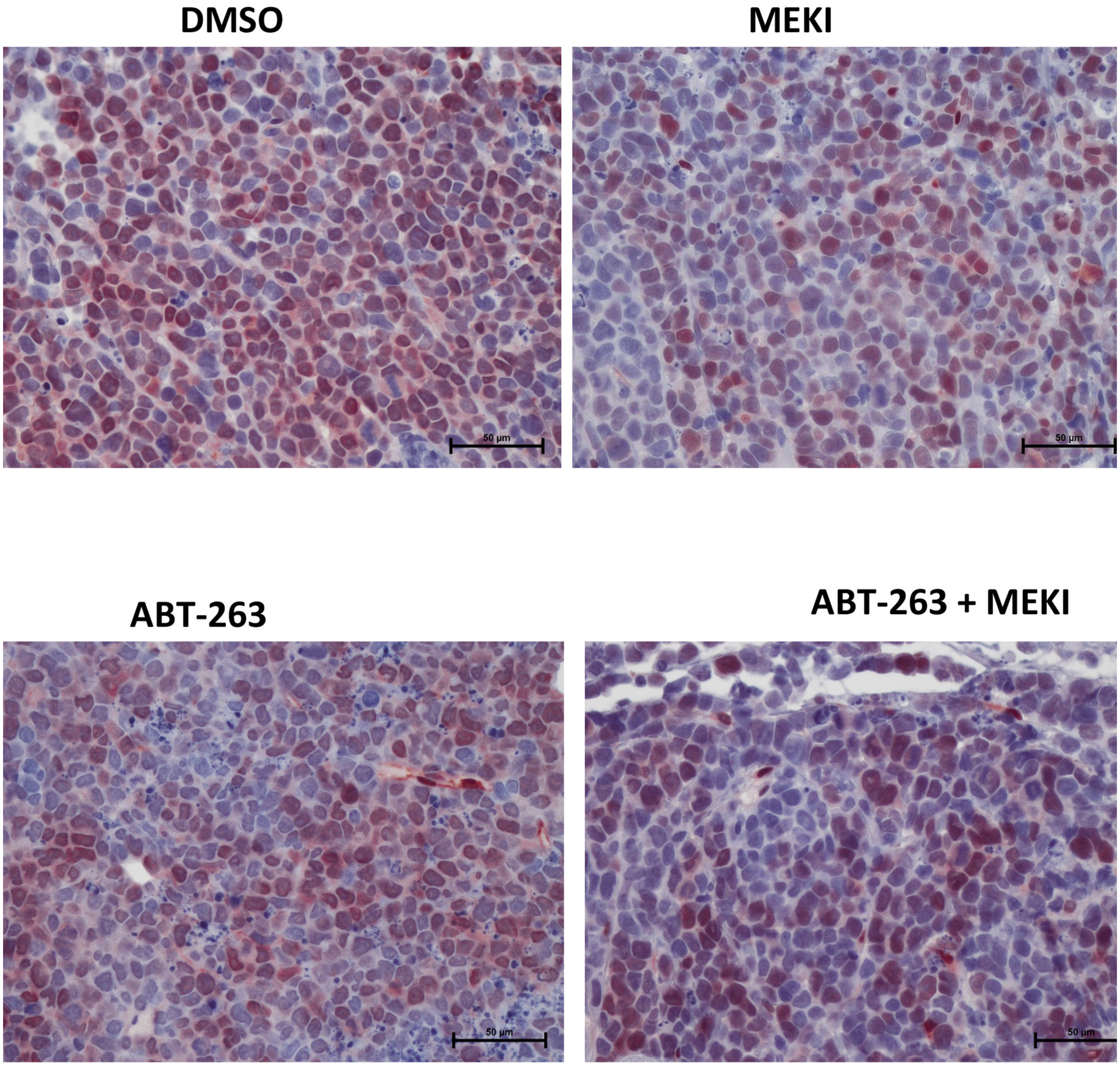

Supplementary Figure S3: An immuno-staining with an anti-phospho-ERK1/2.
